# Insect Neuropeptide Bursicon Homodimers Induce Innate Immune and Stress Genes during Molting by Activating the NF-κB Transcription Factor Relish

**DOI:** 10.1371/journal.pone.0034510

**Published:** 2012-03-28

**Authors:** Shiheng An, Shengzhang Dong, Qian Wang, Sheng Li, Lawrence I. Gilbert, David Stanley, Qisheng Song

**Affiliations:** 1 Division of Plant Sciences, University of Missouri, Columbia, Missouri, United States of America; 2 Shanghai Institute of Plant Physiology and Ecology, Chinese Academy of Sciences, Shanghai, China; 3 Department of Biology, University of North Carolina, Chapel Hill, North Carolina, United States of America; 4 USDA/Agricultural Research Service, Biological Control of Insects Research Laboratory, Columbia, Missouri, United States of America; Duke University Medical Center, United States of America

## Abstract

**Background:**

Bursicon is a heterodimer neuropeptide composed of two cystine knot proteins, bursicon α (burs α) and bursicon β (burs β), that elicits cuticle tanning (melanization and sclerotization) through the *Drosophila* leucine-rich repeats-containing G protein-coupled receptor 2 (DLGR2). Recent studies show that both bursicon subunits also form homodimers. However, biological functions of the homodimers have remained unknown until now.

**Methodology/Principal Findings:**

In this report, we show in *Drosophila melanogaster* that both bursicon homodimers induced expression of genes encoding antimicrobial peptides (AMPs) in neck-ligated adults following recombinant homodimer injection and in larvae fat body after incubation with recombinant homodimers. These AMP genes were also up-regulated in 24 h old unligated flies (when the endogenous bursicon level is low) after injection of recombinant homodimers. Up-regulation of AMP genes by the homodimers was accompanied by reduced bacterial populations in fly assay preparations. The induction of AMP expression is via activation of the NF-κB transcription factor Relish in the immune deficiency (Imd) pathway. The influence of bursicon homodimers on immune function does not appear to act through the heterodimer receptor DLGR2, i.e. novel receptors exist for the homodimers.

**Conclusions/Significance:**

Our results reveal a mechanism of CNS-regulated prophylactic innate immunity during molting via induced expression of genes encoding AMPs and genes of the Turandot family. Turandot genes are also up-regulated by a broader range of extreme insults. From these data we infer that CNS-generated bursicon homodimers mediate innate prophylactic immunity to both stress and infection during the vulnerable molting cycle.

## Introduction

Insect growth and development involve a series of molts during which the old cuticle is digested while a new cuticle is formed and the remnant discarded (ecdysis) [1]. When insects shed this remnant, a new, soft and untanned cuticle is exposed that is vulnerable to injury and attack [2], [3]. Insects must quickly tan (melanize and sclerotize) newly formed, soft cuticle after each molt to survive. In *Drosophila* the neurohormone bursicon, composed of two heterodimer cystine knot proteins, bursicon α (burs α) and bursicon β (burs β), mediates the tanning process in newly eclosed adults [4], [5] via the *Drosophila* leucine-rich repeats-containing G-protein-coupled receptor (DLGR2), encoded by *rickets* (*rk*) [6], [7]. DLGR2, once activated, elicits the cAMP/PKA signaling pathway [4], [5], leading to activation of tyrosine hydroxylase, a key enzyme responsible for tanning agent synthesis [8]. In addition, bursicon heterodimer also regulates integumentary structure development, adult eclosion, and wing expansion and maturation, all multi-step processes [9]–[12].

Recent studies show that in addition to forming the heterodimer responsible for cuticle tanning [4], [5] and wing maturation in newly emerged adults [9]–[12], bursicon subunits also form burs α−α and burs β−β homodimers [4]. However, neither burs α−α nor burs β−β homodimers induce tanning and other endocrine actions established for the heterodimer. Thus, the biological functions of burs α−α and burs β−β homodimers remain unknown.

While traditionally regarded as a developmental hormone, two reports suggest additional bursicon roles in insect biology. Burs α and β proteins are released from the CNS before the initiation of the pupal-adult molting cycle [13] and they have been localized in the CNSs of all larval stages and in prepupae of *Drosophila* [4], [5] and the house fly, *Musca domestica* [14], indicating that both bursicon subunits are present throughout post-embryonic development. Secondly, treating neck-ligated fruit flies (a classical procedure that effectively separates brain endocrine factors from the rest of the body) with recombinant bursicon (r-bursicon) heterodimer influenced the expression of 87 genes, most of which act during tanning and wing maturation [15]. However, seven of these are immune response genes, suggestive of a novel bursicon function in *Drosophila* juvenile and adult immunity. Because invertebrates utilize innate, but not adaptive, immunity [16], [17], these data led us to hypothesize that bursicon homodimers mediate expression of innate immunity genes that encode anti-microbial proteins (AMPs). Reasoning that molting periods are times of heightened vulnerability to potential injury and attack, expression of AMPs during molting would be a form of prophylactic innate immunity that operates to prevent, rather than respond to, infection. Here we report on outcomes of experiments that strongly support our hypothesis and demonstrate a novel mechanism of CNS regulation of insect innate immunity.

## Results

### Bursicon forms homodimers as well as the classical heterodimers

**Figure 1:**
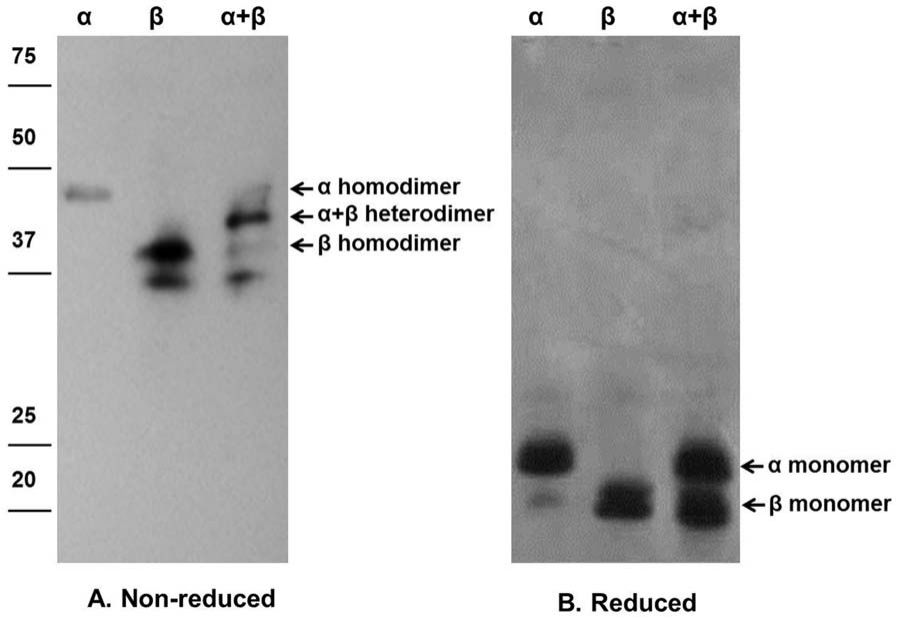
Western blot analysis of r-bursicon proteins in non-reduced (A) and reduced (B) SDS-PAGE identified with an anti-His-Tag antibody. The positions of the r-burs α−β heterodimer, burs α−α and burs β−β homodimers are indicated. Lanes annotated α, β and α+β indicate separate expression of burs α and burs β and co-expression of α+β. The monomers are not present under non-reducing conditions (A). Numbers on far left indicate positions of molecular weight standards.

We expressed r-bursicon subunits in mammalian HEK293 cells, purified the proteins and confirmed their identity. When expressed as individual subunits, they form burs α−α and burs β−β homodimers. We recognized the homodimers because the molecular size of burs α or burs β doubled in the non-reduced gel when compared to the sizes in the reduced gel (Fig. 1). This result is consistent with what had been reported by Luo et al. [4]. When co-expressed, most burs α and burs β subunits (>80%, based on Western blot densitometry) form the bursicon α−β heterodimer while the remaining portion form burs α−α and burs β−β homodimers (Fig. 1). We confirmed the tanning activity of the r-burs α−β heterodimer by injection into neck ligated flies (Fig. S1). Whereas the control, burs α−α and burs β−β homodimer injections did not influence tanning, the r-burs α−β heterodimer and homogenates of the CNS from newly emerged flies (a positive control [15]) elicited tanning beginning 30 min post-treatment (pt).

### Burs α−α and burs β−β homodimers mediate expression of immunity-conferring genes

**Figure 2:**
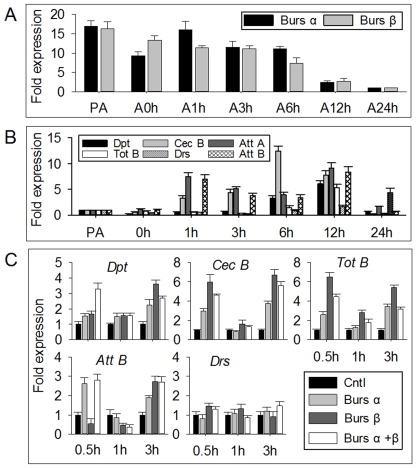
Transcription expression profiles of burs α and burs β subunits and AMP genes in pharate and newly-emerged adults. qPCR analysis (A) of bursicon α and β gene expression in pharate (PA) and eclosed adults (A) 0–24 h after eclosion; qPCR analysis (B) of AMP gene expression in pharate and eclosed adults 0–24 h after eclosion; Transcription expression (**C**) of AMP genes in 24 h-old adults injected with blank vector transfected sample, r-burs α−α homodimer, r-burs β−β homodimer and r-burs α−β heterodimer, respectively.

We registered an inverse correlation between the reduction in bursicon transcript levels (associated with release of bursicon) (Fig. 2A) [13] and a significant increase in the transcript levels of several representative AMP genes [*Diptericin (Dpt)*, *Cecropin B (Cec B)*, *Attacin A (Att A)*, *Turandot B (Tot B)*, *and Attacin B (Att B)*], but not *Drosomycin (Drs)*, a Toll pathway specific marker (Fig. 2B) in adults during the first 12 h after emergence. We infer that the increase of AMP transcript levels in newly emerged flies is regulated by burs α−α and burs β−β homodimers. After the 24 h-old files, which displayed low levels of bursicon transcripts and AMP genes, were injected with the r-burs α−α and burs β−β homodimers, the transcript levels of the representative AMP genes except *Drs* were up-regulated (Fig. 2C), demonstrating a role for bursicon homodimers in mediating AMP gene transcription *in vivo*.

**Figure 3:**
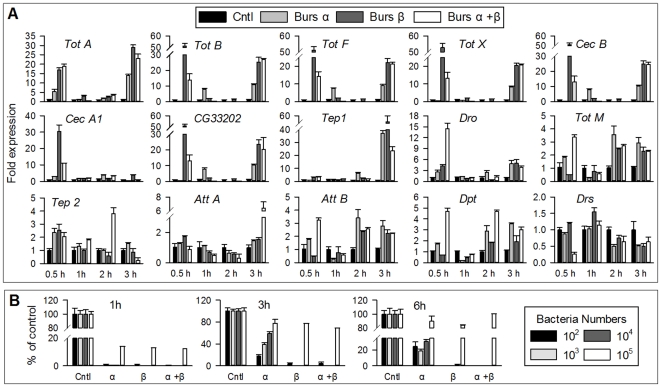
Burs α−α and burs β−β treatments induced expression of AMP genes and suppression of bacterial growth in adults. Separate groups of adult flies were neck-ligated immediately after emergence and at 1 h post-ligation injected with 60 ng/0.5 µl of r-burs α−β, burs α−α, or burs β−β. The control flies were injected with the purified cell culture transfected with blank pcDNA 3.1 vector. After the indicated incubation periods, expression of the AMP genes was determined by qPCR. (A) AMP transcript levels in wild type adults. For bacterial inhibition assay (B), neck-ligated wild type flies were injected with r-burs α−β heterodimer, burs α−α homodimer, burs β−β homodimer or blank vector transfected sample, respectively, homogenized and centrifuged for 15 min at 16,000 g. The resulting supernatants were challenged with indicated titers of *E. coli* for 6 h before plating for colony count. The histograms show the means ± SEM, n = 3 biologically independent experiments.

We then used the r-burs α−α and burs β−β homodimer proteins to further test our hypothesis that bursicon homodimers mediate the expression of immunity-conferring genes in neck-ligated adults (a classic method to prevent the endogenous bursicon release) (Fig. 3). The qPCR results show that relative to blank pcDNA 3.1 vector-treated controls, expression of eight genes [*Turandot A (Tots A)*, *Tot B*, *Tot F and Tot X*, *Cec B*, *Cec A1*, *CG33202 and Thioester-containing protein 1(Tep 1*)] was substantially (>19-fold) up-regulated following injection of burs α−α and burs β−β homodimers into neck-ligated wild type adults 0.5 to 3 h post injection (Fig. 3A). Six other genes [*Drosocin* (*Dro*), *Tot M*, *Tep 2*, *Att A*, *Att B* and *Dpt*] were up-regulated to a lesser extent, by ≥2-fold, while the expression of *Drs*, a Toll pathway specific marker and a control in these studies, was not influenced by burs α−α and burs β−β homodimer injection. These data indicate that burs α−α and burs β−β homodimers mediate expression of several immunity-conferring genes in immunologically naïve adult *Drosophila*, possibly via the Imd pathway because *Dpt*, a Imd pathway marker, was upregulated by the homodimers. We note that these data do not address the central issue of whether the up-regulated genes constitute prophylactic immunity.

### Burs α−α and burs β−β homodimer-induced gene transcripts are translated into AMPs

We next considered whether the up-regulated gene transcripts were translated into operational AMPs. Neck-ligated flies were injected with r-burs α−α and burs β−β homodimer, or r-burs α−β heterodimer or blank vector transfected sample (control), collected and homogenized after selected time periods. The resulting supernatants were incubated with bacteria for 6 h (Fig. 3B for Gram^−^ *E. coli* and Fig. S2 for Gram^+^ *M. luteus*). After plating and incubating the mixtures, bacterial colonies were counted. Incubations in the presence of 10^2^, 10^3^ and 10^4^ *E. coli* cells/fly equivalent showed that most bacterial cells were killed at 1 h pt, with some bacterial growth in the presence of 10^5^ *E. coli* cells. The inhibitory effect was reduced at 3 and 6 h pt, which we ascribe to AMP turnover. qPCR analyses (Fig. 3A) and antibacterial assays (Fig. 3B) revealed a general pattern that the r-burs β−β homodimer induces gene expression and inhibits bacterial proliferation quicker and more efficiently than does r-burs α−α homodimer, possibly due to differences in their receptor affinities. The effect of the heterodimer on AMP transcript level and antibacterial activity may result from a small quantity of homodimers remaining in the co-transfected sample as seen in Fig. 1. We also recorded a similar, albeit smaller, inhibitory effect on Gram^+^ bacteria (Fig. S2). These data demonstrate that the induced AMPs effectively killed bacterial cells, except in the case of the 10^5^ samples, in which the induced AMPs provided insufficient responses to overwhelming bacterial populations. These results establish a functional link between increased AMP gene transcripts and effective prevention of bacterial infection.

### Bursicon homodimers mediate expression of genes encoding AMPS in isolated larval FB

**Figure 4:**
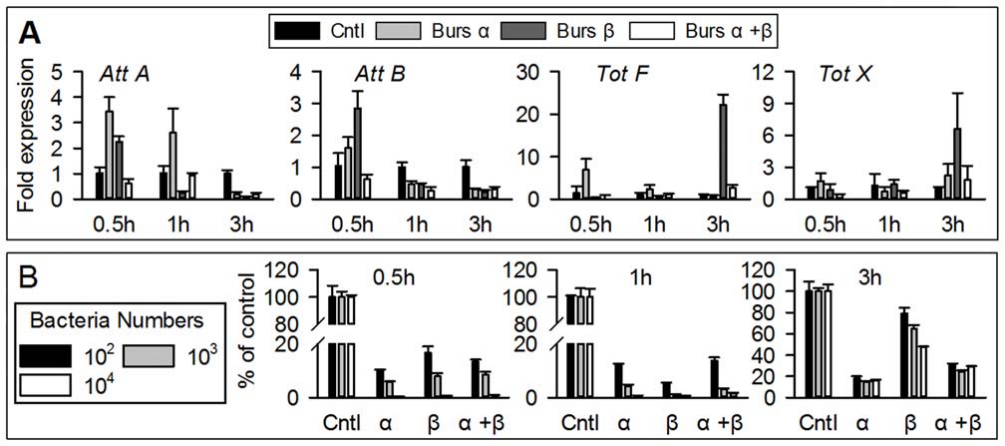
Burs α−α and burs β−β treatments induced expression of AMP genes and suppression of bacterial growth in larva FB. FB was dissected from early wondering 3^rd^ instar larvae and incubated with r-burs α−α or burs β−β homodimer or r-burs α+β heterodimer for 0.5, 1 and 3 h. The control group received the blank vector transfected sample. After incubation, RNA was extracted for qPCR analysis of 4 representative AMP genes (A). For bacterial inhibition assay, FB was homogenized and centrifuged at 16000 g for 20 min at 4°C. The resulting supernatants were challenged with indicated titers of *E. coli* for 6 h before plating for colony count (B). The histograms show the means ± SEM, n = 3 biologically independent experiments.

Most AMPs are synthesized in the FB of *Drosophila* [16] and both bursicon subunits are expressed in larval, pupal and adult stages [4], [5], [8]. We investigated the hypothesis that burs α−α and burs β−β homodimers would also induce expression of AMPs in an *in vitro* FB bioassay. Isolated FBs from early wandering third instar larvae were incubated in the presence of burs α−α or burs β−β homodimers and the effects measured. Burs α−α and burs β−β homodimers up-regulated the transcript levels of four representative genes (*Att A*, *Att B*, *Tot F* and *Tot X*) by 2–20 fold (Fig. 4A). As was seen in whole fly preparations, burs α−α or burs β−β homodimer-treated FB preparations similarly eliminated bacterial cells (Fig. 4B). Our *in vitro* FB assays therefore confirmed the *in vivo* bioassay results in adults, and, most importantly, demonstrate for the first time that burs α−α or burs β−β homodimers exerts a novel function in the prophylactic immunity of both juvenile and adult *Drosophila*.

### Burs α−α or burs β−β homodimers do not act through the heterodimer receptor DLGR2

**Figure 5:**
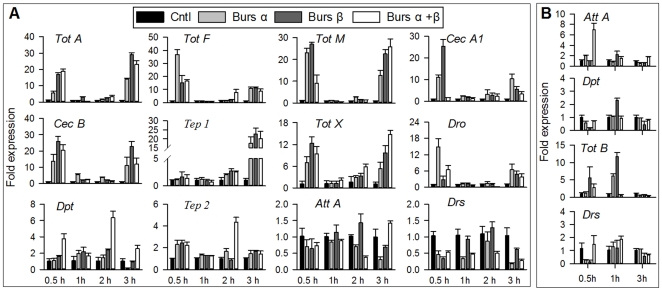
AMP transcript levels in *rk^4^* mutant. Newly emerged adult flies were neck-ligated immediately after emergence and at 1 h post-ligation injected with r-burs α−β heterodimer, burs α−α homodimer, burs β−β homodimer or blank vector transfected sample, as described in Figure 3. RNA was extracted for qPCR analysis of 11 representative genes (A). Larva FB from the mutant was also used to assay the effect of mutation on burs α−α and burs β−β homodimer induced AMP expression (B). The histograms show the means ± SEM, n = 3 biologically independent experiments.

Because the *Drosophila* bursicon heterodimer acts through the G-protein coupled receptor DLGR2 [4]–[7], we next investigated whether the bursicon homodimer proteins induce expression of immunity-conferring genes through DLGR2, encoded by the *rk* gene [6], [7]. We used the loss-of-function mutant line *rk^4^*, to determine whether burs α−α or burs β−β homodimers operate via DLGR2. *rk^4^* flies were injected with r-bursicon homodimers, and the transcript levels of 10 representative genes encoding AMPs (*Tot A*, *Tot F*, *Tot M*, *Cec A1*, *Cec B*, *Tep 1*, *Tot X*, *Dro*, *Dpt* and *Tep 2*) were assessed. These treatments with the r-bursicon homodimers led to increased expression of all 10 genes in adults (Fig. 5A) and of three genes (*Att A*, *Dpt and Tot B*) in larval fat body (Fig. 5B) as just described in wild type adults (Fig. 3A). As seen in the wild type adults, none of the bursicon homodimers influenced *Drs* expression over the 3-h course of the experiments with the mutants. These data support our assertion that the r-burs homodimers do not influence expression of genes encoding AMPs via DLGR2 and raise the possibility of novel receptors for burs α−α and burs β−β homodimers. We note that *rk^4^* mutants may be hypomorphs with reduced, rather than completely blocked, expression of DLGR2 [13]. Because expression of AMP-encoding genes was not reduced in the mutant line compared to similarly treated wild type flies, it appears to us that burs α−α or burs β−β homodimers do not act through DLGR2.

### Burs α−α and burs β−β homodimers activate the Imd pathway by activating Relish

**Figure 6:**
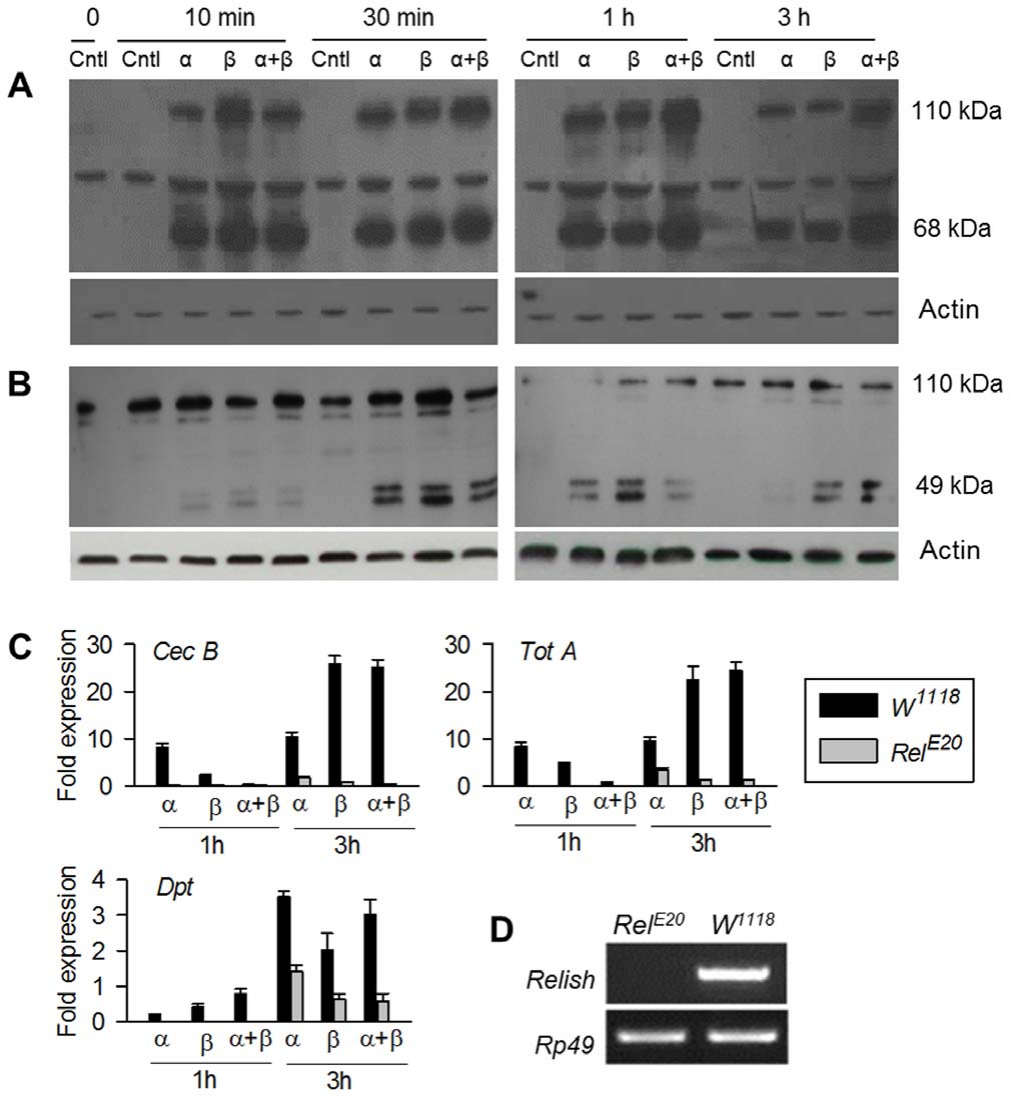
Western blot analysis of NF-κB transcription factor Relish activation in wild type larvae and adults. (A) Neck ligated flies were treated with indicated r-burs α−β, burs α−α, burs β−β or blank vector transfected sample. After the indicated incubation periods the flies were homogenized and then subjected to Western blot analysis using anti-Relish polyclonal antibodies. Bands labeled actin show protein loading controls. (B) Third instar larval fat body preparations were treated with the indicated bursicon proteins. After incubation for the indicated times the FB preparations were homogenized and subjected to Western blot analysis using anti-Relish monoclonal antibody. (C) qPCR analysis of 3 representative genes in the wild type and Relish mutant *Rel^E20^* flies. (D) RT-PCR analysis shows the presence of Relish transcripts in the wild type controls, but not the mutant *Rel^E20^*. The histograms show the means ± SEM, n = 3 biologically independent experiments. Actin loading controls are present in the bottom of A and B.

Neither the burs α−α or burs β−β homodimer influenced the transcript level of *Drs*, a Toll pathway-specific marker gene, but both strongly up-regulated the transcript level of *Dpt*, an Imd pathway marker. These data raise the possibility that r-burs α−α or burs β−β homodimers individually influence the Imd pathway, which in *Drosophila* is activated in response to Gram (^−^) bacteria [16]. The *Drosophila* NF-κB-like transcription factor, Relish, is activated by signal-driven, Dredd-catalyzed endoproteolytic cleavage into two fragments, an N-terminal DNA-binding Relish homology fragment which migrates into the nucleus and a C-terminal stable IκB-like fragment that remains in the cytosol [16], [17]. We injected neck-ligated adult flies with r-burs α−α or burs β−β homodimers and after the indicated time periods prepared protein samples for Western blot analysis using an anti-Relish N-terminal sequence polyclonal antibody [18]. Burs α−α or burs β−β protein treatments led to rapid Relish activation (Fig. 6A, seen as the 68 kDa active fragment) [18], the r-burs β−β homodimer more potent in the first 10 min and the r-burs α−α homodimer more so at 1 h pt. Relish activation waned by 3 h pt. We also analyzed the r-bursicon homodimer activation of Relish in larval FB preparations using an anti-Relish C-terminal sequence monoclonal antibody. Burs α−α or burs β−β homodimers activated Relish as just described for adults (Fig. 6B, seen as the C-terminal 49 kD inactive fragment). We note the bursicon heterodimer also activates Relish, which we ascribe to small amounts of homodimers present in the heterodimer preparation.

To verify the activation of Relish by burs α−α or burs β−β homodimers as shown in Western blot analysis (Fig. 6A and B), the loss of function mutant (*Rel^E20^*) flies were neck-ligated immediately after emergence, injected with r-bursicon homodimers and analyzed for their response. As shown in Fig. 6C, the r-bursicon homodimer-induced expression of three representative genes did not occur in mutant flies, confirming that burs α−α or burs β−β homodimers elicited AMP expression is via Relish activation. It should be noted that the Relish transcript was not detectable in this mutant (Fig. 6D). On the basis of these data we postulate that bursicon homodimers influence the expression of immune genes via the Imd pathway by activating Relish.

## Discussion

**Figure 7:**
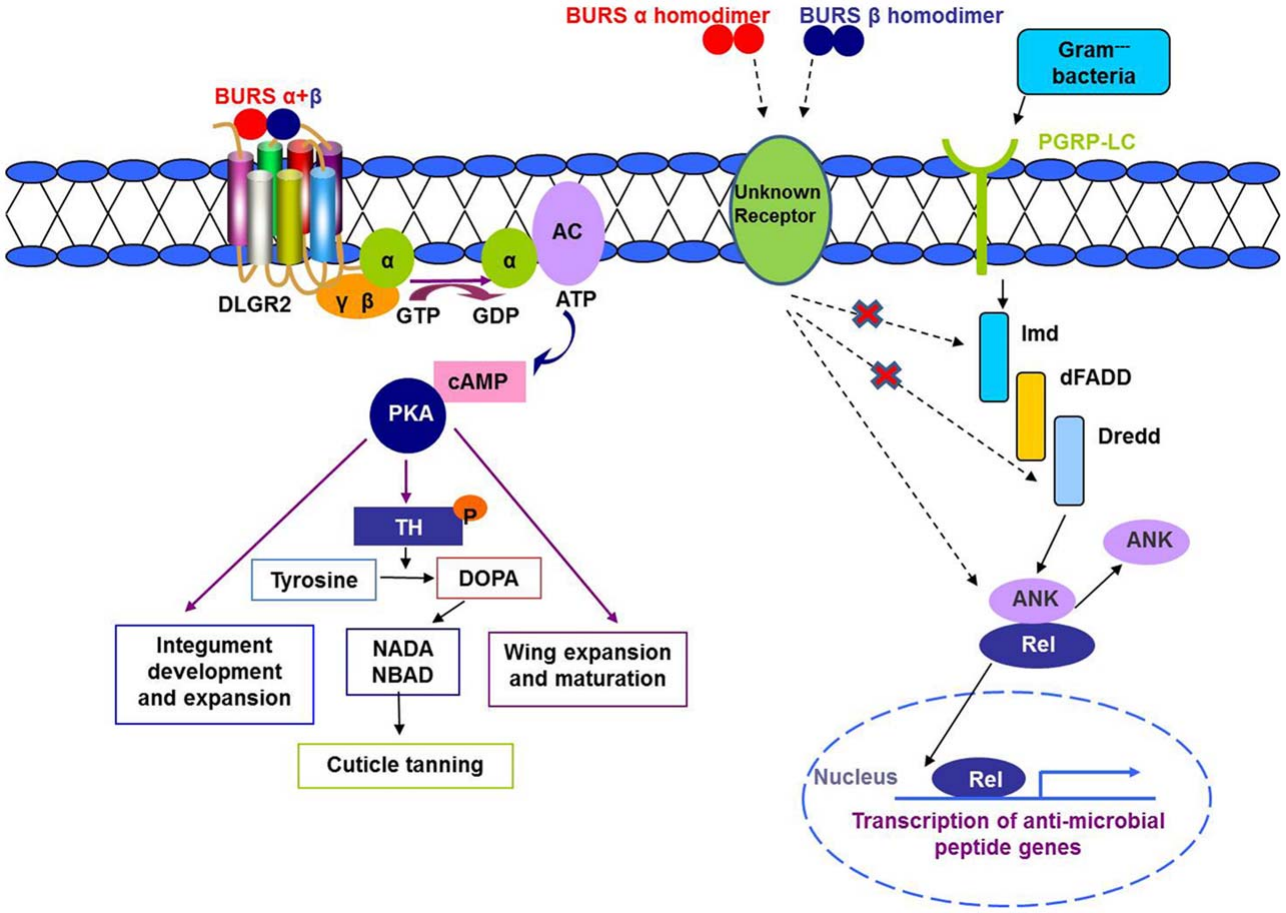
A partly hypothetical model of bursicon signaling pathways, showing the known interaction between burs α−β heterodimer and its receptor, DLGR2, and interactions between burs α−α and burs β−β homodimers and a hypothesized receptor(s) leading to the transcription of AMP genes. The model also shows the Imd pathway and the position of Relish. Once activated, Relish translocates into the nucleus, where it promotes transcription of genes encoding AMPs.

The data in this paper support our hypothesis that the CNS influences innate immunity via secretion of a neurohormone and thus expands the biological roles of bursicon beyond cuticle tanning and wing expansion. Several points are germane. First, the expression patterns of genes encoding burs α and burs β subunits and six AMPs in untreated pharate and newly-eclosed adults appear in strong inverse correlation. Second, injection of r-burs α−α or burs β−β homodimers to the 24 h-old files, which displayed low levels of bursicon transcripts and AMP genes, up-regulates AMP genes, demonstrating a role for bursicon homodimers in mediating AMP gene transcription *in vivo*. Third, bursicon acts in the novel homodimer configuration. Fourth, the bursicon homodimers induce expression of genes encoding AMPs via the activation of the NF-κB transcription factor Relish. And fifth, burs α−α or burs β−β homodimers do not appear to regulate their effects on the immune system via the established heterodimer receptor, DLGR2. Sequence analysis revealed that the burs α and β subunits have no similarity to bacterial cell wall proteins, which bind the peptidoglycan recognition protein (PGRP) to activate immune responses. Although PGRP binding to bursicon has not been experimentally ruled out, we infer that bursicon homodimers do not act through the PGRP. Hence, bursicon homodimers activate components in the Imd signaling pathway, downstream of PGRP, but upstream of Relish. Future studies will focus on the identification of the novel receptor(s) involved in the action of bursicon homodimers on the immune system (Fig. 7). Despite the preponderance of work on mammalian immunity [19], exactly how the CNS controls inflammation and the immune response is not understood completely [20]. Perhaps the *Drosophila* model with its abundant genetic repertoire will help solve this ‘ancient problem’.

Bursicon is a member of the cystine knot protein family, which includes vertebrate glycoprotein hormones, growth factors, mucins, and bone morphogenetic protein antagonists [2]. All these hormones, including bursicon, share a common structural feature of a α-subunit and a β-subunit which form the physiologically operational heterodimers. Some, such as the placental chorionic gonadotropin, also form homodimers to execute a different physiological function. Luo et al. [4] show that each of the bursicon subunits forms a homodimer, shown *in vitro* in *Drosophila* by Western blot and *in vivo* in several insect species including *Drosophila* by immunocytochemistry [2], but the function of the homodimers was, until now, unknown. Here, we identify one role for the bursicon homodimers as mediators of the prophylactic expression of genes encoding AMPs.

We also note that bursicon homodimers induce expression of several *turandot* and *Tep1* genes, markers for the JAK/STAT pathway. Since the transcriptional regulation of the JAK/STAT pathway requires inputs from the Imd pathway [16], up-regulation of *turandot* and *Tep1* genes could result from up-regulation of the Imd pathway. The biological significance of these gene products extends beyond anti-microbial actions to the generalized responses to extreme stressors [21]. While other stress-responsive proteins, such as heat shock proteins, act within cells, the Turandot proteins are secreted into the hemolymph following a variety of stress experiences [22]. Like developmental and reproductive events in many animals, molting produces actual and potential stresses in insects, including increased energy demands (producing reactive oxygen species), water loss, ion imbalances, injury and infection. We conclude that the expression of general stress-responsive genes could be an important adaption during the highly susceptible time of the molting cycle.

## Materials and Methods

### Drosophila stocks

*W^1118^* was used as the wild type strain. The *rk^4^* and *Rel^E20^* mutants were ordered from the Bloomington *Drosophila* stock center. The fly stocks were maintained on artificial blue culture medium (Fisher Scientific) at 20°C under constant darkness.

### r-Bursicon protein expression

The r-bursicon proteins were expressed in HEK293 cells and purified as described [15]. Briefly, CNSs (fused thoracic/abdominal ganglia) were isolated from pharate adults in Ringers' solution (3.6 mM NaCl, 54.3 mM KCl, 8.0 mMCaCl2, 28.3 mM MgCl2). Pharate adults express high levels of burs α and burs β transcripts [4], [5], [14]. Total RNA was extracted using Trizol reagent (Invitrogen) according to the manufacturer's instructions. The burs α and burs β open reading frames were amplified using a gene specific forward primer with a *Xho*I restriction site and a reverse primer with a *BamH*1 restriction site (burs α: forward primer 5′-CTCGAGATGCTGCGCCACCTGCTCCG-3′; reverse primer 5′-GGATCCTTGCAGAGCAATGCGTCCGGA-3′. Burs β: forward primer 5′-CTCGAGATGCATGTCCAGGAACTGCT-3′; reverse primer 5′-GGATCCACGTGTGAAATCGCCACATT-3′). The amplified fragments were inserted into the PGEM-T-Easy vector (Promega), and sequenced again for confirmation of correct insertions.

The pcDNA3.1/*myc*-His (−) B expression vector with a C-terminal peptide containing a polyhistidine metal-binding tag, was used to express the r-bursicon in HEK293 cells (American Type Culture Collection, Manassas, VA). The *Drosophila* burs α and burs β genes were retrieved from the PGEM-T-Easy vector by digestion with BamHI and XhoI and ligated into the pcDNA3.1/*myc*-His (−) B expression vector predigested with XhoI and BamH1. The recombinant vector was sequenced to confirm correct insertions. The recombinant pcDNA3.1/*myc*-His (−) B plasmids (2 µg) were transfected into mammalian HEK293 cells either individually or simultaneously using the SatisFection™ Transfection Reagent (Strategene). Control cells were transfected with the blank vector (containing no burs α or burs β cDNA insert). After 16 h, the serum-free DMEM cell culture medium was replaced with fresh medium supplemented with 10% fetal bovine serum and the transfected cells were incubated for another 24 h. The medium was replaced again with serum-free DMEM and cultured. After 48 h, the medium was collected and centrifuged at 2000×g to remove cell debris.

The expressed r-bursicon proteins were purified using Ni-NTA His-bind resin (QIAGEN) and separated by 12.5% SDS-PAGE (no reducing agent was added to non-reducing gel) and their identities confirmed by Western blot (see the following section for detail procedure) using a His-tag antibody (Sigma, 1∶2000 dilution) prior to use in bioassays.

### r-Bursicon injections

Newly emerged flies were neck-ligated immediately after emergence and allowed a 1 h waiting period to ensure a complete ligation (no sign of cuticle tanning). r-Burs α−α and burs β−β homodimers or r-burs α−β heterodimer proteins were injected into thorax-abdomens of neck-ligated flies with untanned cuticle in 0.5 µl (60 ng protein, drawn from Luo et al. [4]) volumes using a microinjection system equipped with a hand-calibrated pulled glass needle, diameter ∼4 microns. Control groups were injected with the blank-vector transfected sample (purified using the same procedure as for r-burs proteins). For analysis the response of unligated flies to r-burs α−α and burs β−β homodimers, the 24 h old flies (endogenous bursicon level is low) were injected with r-bursicon homodimers as described. After the indicated incubation periods, the flies were processed for RNA extraction for qPCR or protein extraction for Western blot analysis.

### Larval fat body incubation

Fat body (FB) was dissected from early wondering 3^rd^ instar larvae under Ringer's solution and immediately placed in insect Grace's medium. FBs were incubated with 50 µl fresh medium containing 1 µl (100 ng/µl) of r-burs α−α or burs β−β homodimer or r-burs α−β heterodimer for 0.5, 1 and 3 h. The control group received the blank vector transfected sample. After incubation, FB was processed for RNA and protein extraction as described above. For bacterial inhibition assays, treatment and control groups were homogenized on ice and centrifuged at 16000 g for 20 min at 4°C. Supernatant was collected for bacterial inhibition assay (see the following section).

### qPCR analysis of AMP genes

Total RNA was extracted from the treated and control flies or larvae FBs using the Trizol reagent (Invitrogen) according to the manufacturer's instructions. First-strand cDNA was synthesized from 2 mg DNAse-treated total RNA using an oligo-dT_20_ primer and superscript™ III reverse transcriptase as the enzyme (Invitrogen). Gene specific primers (Table S1) were used for qPCR amplification of AMP genes. qPCR amplification and analysis were carried out on an Applied Biosystems (ABI) 7500 Fast Real-Time PCR System. The final reaction volume was 25 µl using ABI SYBR green Supermix (ABI). The PCR program was: hold at 95°C for 10 min and then at 95°C for 15 seconds and 60°C for 1 min, repeating 40 cycles. The specificity of the SYBR green PCR signal was further confirmed by a melting curve analysis and agarose gel electrophoresis.

### Western blot analysis of Relish activation

Neck-ligated adult flies were injected with r-burs α−α homodimer, r-burs β−β homodimer, r-burs α−β heterodimer or blank vector-transfected sample (control) as described above, and then proteins were extracted, quantified and subjected to SDS page (12.5%). Proteins separated in gels (15 µg/lane) were transferred onto PVDF membranes using a BioRad Trans-blot SD Semi-Dry Transfer Cell. After protein transfer, the PVDF membranes were blocked with 5% non-fat dried milk in a Tris-buffered saline (50 mM Tris-HCl, 150 mM NaCl, 1 mM EDTA, 0.1% Tween 20, pH 7.5) (TBST) for 1 h, and then incubated overnight at 4°C with the anti-Relish N polyclonal antibody (1∶2000; gift from Prof. Svenja Stöven, Umeå University, Sweden) in the blocking solution. After incubation with the primary antibodies, the membranes were washed 3 times with TBST, 10 min each, and then incubated with HRP-conjugated goat anti-mouse secondary antibody (diluted at 1∶2000 in the blocking solution) for 90 min at room temperature. After washing with TBST for 30 min, the membranes were treated for 1 min with SuperSignal® West Pico chemiluminescent substrate (PIERCE) and the immunoreactive proteins were visualized by exposing an x-ray film to the membrane.

Similarly, Western blot analysis of Relish activation in larva FB preincubated with r-burs α−α homodimer, r-burs β−β homodimer, r-burs α−β heterodimer or blank vector-transfected sample (control) was also performed using the anti-Relish C-terminus monoclonal antibody (mAb) with 1∶1000 dilution (the mAb was purchased from the Hybridoma Bank, University of Iowa). Anti-actin antibody was used to ensure that equal amount of protein was loaded in each lane.

### In vitro antibacterial assay

Supernatants from the r-burs α−α homodimer, r-burs β−β homodimer, r-burs α−β heterodimer or blank vector-transfected sample (control)-treated adult flies or larvae FB were mixed with Gram-negative bacteria *Escherichia coli* or Gram-positive bacteria *Micrococcus luteus* at 10^2^, 10^3^, 10^4^ or 10^5^/fly equivalent. After 6 h incubation at 37°C, the mixtures were plated and bacterial colonies counted after overnight incubation at 37°C.

## Supporting Information

Figure S1 **A neck-ligated bioassay for r-burs tanning activity.** The flies were neck-ligated immediately after emergence. After a 1 h waiting period, the flies with untanned cuticle were injected with purified r-burs α−β, burs α−α or burs β−β (60 ng in 0.5 µl) (experimental groups). Control group received the purified sample transfected with blank pcDNA3.1 plasmid. Sclerotization was assessed visually following the indicated incubation periods and representative flies were photographed at 40× under a Leica MZ16 microscope with apochromatic correction and a Qimaging digital camera.
(TIF)

Figure S2 **Burs α−α and burs β−β homodimer treatments suppressed** ***M. luteus*** **populations in adult fly preparations.** The neck-ligated flies were injected with r-burs α−β, burs α−α, burs β−β or control sample transfected with blank vector for 1, 3 and 6 h, and the resulting supernatant was then challenged with preparations of *M. luteus* for 6 h. The histograms show *M. luteus* colonies (as percentages of challenge doses) recovered from adult fly preparations.
(TIF)

Table S1 (DOCX)
