# Supplementary figures and images for: Insect Neuropeptide Bursicon Homodimers Induce Innate Immune and Stress Genes during Molting by Activating the NF-κB Transcription Factor Relish
Source: PLoS One. 2012 Mar 28;7(3):e34510. doi: 10.1371/journal.pone.0034510 (PMC3314635; doi:10.1371/journal.pone.0034510)

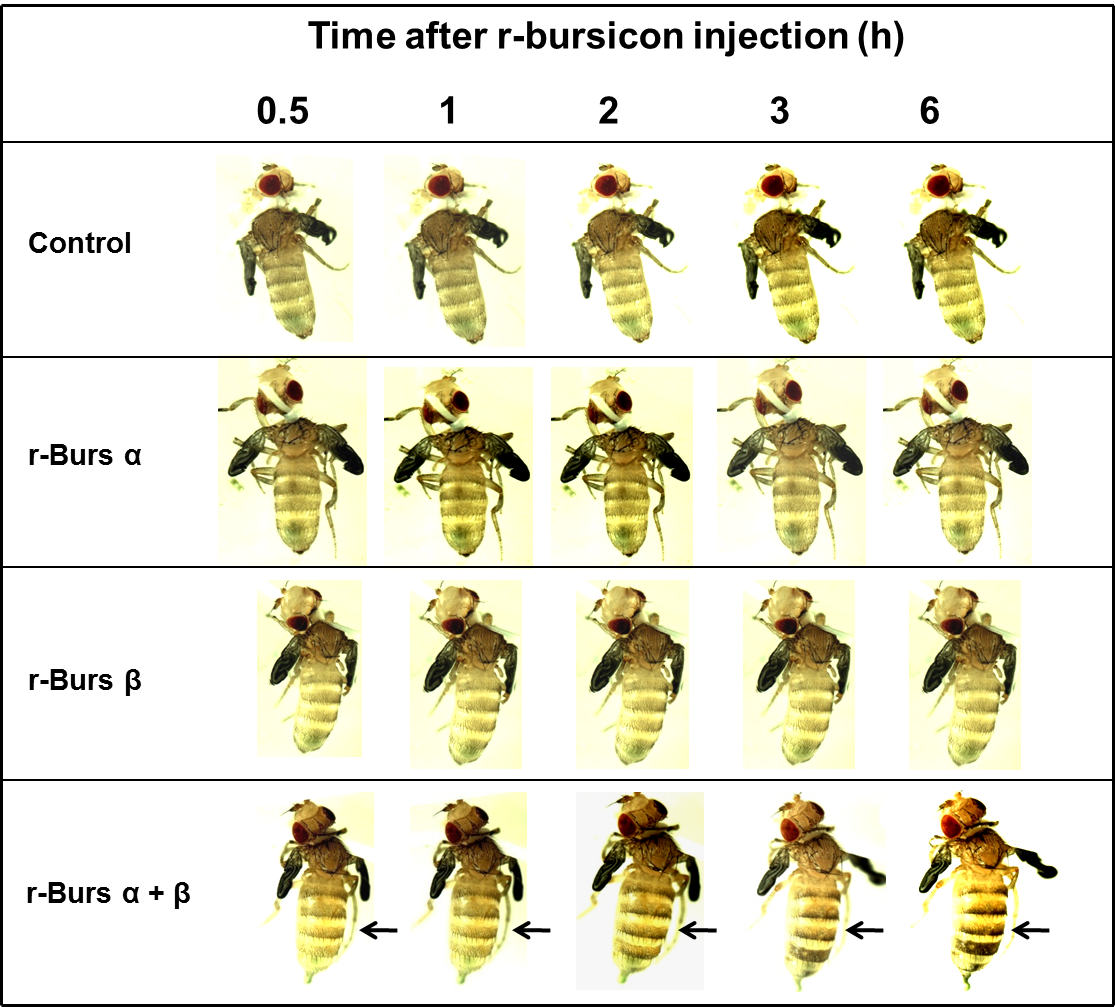

Supplement: Figure S1 — A neck-ligated bioassay for r-burs tanning activity. The flies were neck-ligated immediately after emergence. After a 1 h waiting period, the flies with untanned cuticle were injected with purified r-burs α−β, burs α−α or burs β−β (60 ng in 0.5 µl) (experimental groups). Control group received the purified sample transfected with blank pcDNA3.1 plasmid. Sclerotization was assessed visually following the indicated incubation periods and representative flies were photographed at 40× under a Leica MZ16 microscope with apochromatic correction and a Qimaging digital camera. (TIF) [file pone.0034510.s001.tif]

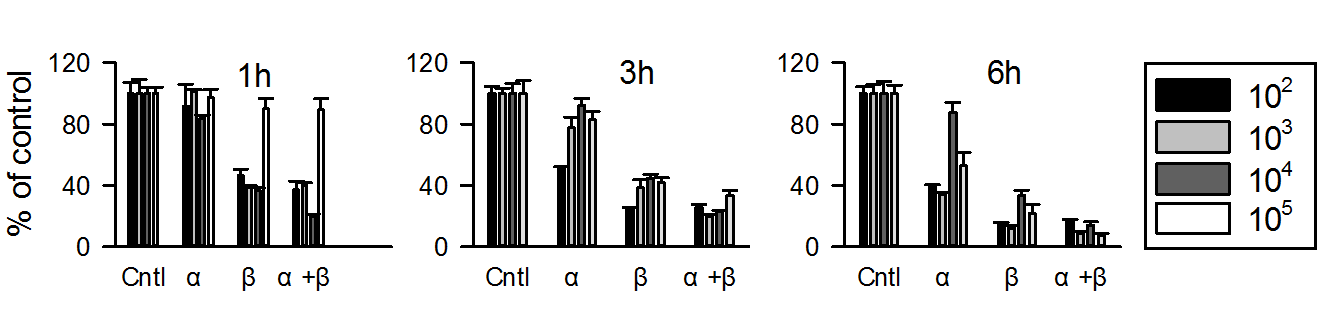

Supplement: Figure S2 — Burs α−α and burs β−β homodimer treatments suppressed M. luteus populations in adult fly preparations. The neck-ligated flies were injected with r-burs α−β, burs α−α, burs β−β or control sample transfected with blank vector for 1, 3 and 6 h, and the resulting supernatant was then challenged with preparations of M. luteus for 6 h. The histograms show M. luteus colonies (as percentages of challenge doses) recovered from adult fly preparations. (TIF) [file pone.0034510.s002.tif]
