# Supplementary material for: Insect Neuropeptide Bursicon Homodimers Induce Innate Immune and Stress Genes during Molting by Activating the NF-κB Transcription Factor Relish
Source: PLoS One. 2012 Mar 28;7(3):e34510. doi: 10.1371/journal.pone.0034510 (PMC3314635; doi:10.1371/journal.pone.0034510)
Supplement: Table S1 — (DOCX) [file pone.0034510.s003.docx]

**Supporting Information Table**

**Table S1. Primers for burs α, burs β and immune-response genes.**

**Gene name Forward sequence Reverse sequence**

*Burs α* 5’-CTTGGCGACGATTGTCAGGT-3 ‘ 5’-GGGACAGAATAGCGAGACGG-3’

*Burs β* 5’-GGCACTGGCGACGAGAACTG-3’ 5’-GGCAGCAGTAGCACTCTTTCAG-3’

*Attacin A* 5’-ACTCAAAGTGGTCCAGTCA-3’ 5’-TCCTGGGAAGTTGCTGTGC-3’

*Attacin B* 5’-CACTCAAAGCGGTCCAGTCA-3’ 5’-GGTTGGCAAACGACCACTC-3

*Cecropin A1* 5’-TCACCATTGGACAATCGGAAGC-3’ 5’-ACATTGGCGGCTTGTTGA-3’

*Cecropin B* 5’-ATGAACTTCAACAAGATCTT-3 5’-TGATTTCAACCTCGAGCGGT-3’

*CG33202* 5’-CCAGTTTGGCTCAAGTGTC-3’ 5’-CTGGTCGGCTATGAAGACG-3’

*Diptericin* 5’-CAGTTCACCATTGCCGTCG-3’ 5’-CATTGTCGCTGGTCCACAC-3’

*Drosocin* 5’-GTTCACCATCGTTTTCCTGC-3’ 5’-AATGGGGCGGGGATGGGAG-3’

*Drosomycin* 5’-ATGATGCAGATCAAGTACTT-3’ 5’-AATTGCTCATGGATTTAGCA-3’

*Tep 1* 5’-ACCGCAAGTGCCTGACCC-3’ 5’-GCGTTCGGTGGCATAGTA-3’

*Tep 2* 5’-GCCCTTAGAAGACGCCGACA-3’ 5’-CCGGGAGTTAAGCTGTCA-3’

*Turandot A* 5’-ATCGTGAGGCTGACAACCTTAGA-3’ 5’-ACCTCCCTGAATCGGAACTC-3’

*Turandot B* 5’-ATCTGTTTCGCACTGCTACT-3’ 5’-TATCCACTTATGAATCTGTC-3’

*Turandot C* 5’-ATGAATGCCTCCATTTCTC-3’ 5’-TTATCCAGTAAAGAGCCTG-3’

*Turandot E* 5’-ATGAATTCCGCACTGAAAAT-3’ 5’-CTAGGCAGATACTCCGTCAAC-3’

*Turandot F* 5’-ATGAATTCCGCACTGAAAT-3’ 5’-CTAGGCAGATACTCCGTCAAC-3’

*Turandot M* 5’-GCTGGGAAAGGTAAATGCT-3’ 5’-ACCAGAATCCGCCTTGTGC-3’

*Turandot X*  5’-ATGGGGCTTTCTATTGGCAG-3’ 5’-TTAGTTATAGTCCTCGATTATG-3’

*Turandot Z* 5’-GCTTTGTTCTGGCAGTGCT-3’ 5’-ACATTGTCAGGAACGGCATCA-3’
